# Tetany Following Thyroid Operations

**Published:** 1931

**Authors:** Duncan Wood

**Affiliations:** Surgeon, Bristol General Hospital


### TETANY FOLLOWING THYROID OPERATIONS.

BY

DUNCAN WOOD, F.R.C.S.,

*Surgeon, Bristol General Hospital.*

---

TETANY following thyroid operations may be a rare complication. Yet Crile [1] states that every surgeon who has had any wide experience with thyroidectomy is but too familiar with the symptoms of parathyroid deficiency. He reports on five cases. Dunhill [2] has had two cases. Von Eiselsberg [3] reports thirteen cases in 1,157 operations for goitre.

In England very little attention has been given by the clinician to this complication, in spite of much experimental work. Of the textbooks, some ignore it, others merely say it is rare ; one book goes so far as to say that it is practically unknown after operations properly performed. This is not very helpful to a surgeon when faced with a case. It is possible that this outlook has prevented some surgeons from publishing their cases. It may also be the reason why cases have been described, not by the operator, but by a medical colleague. The fact remains that tetany, associated with goitre operations, does occur. It is therefore in the interest of the patient to discuss its prevention and treatment.

The cases about to be recorded number five. They occurred among 425 thyroidectomies. These five cases followed goitre operations, at the Bristol General Hospital, during the last seventeen years.

The years were 1914, 1916, 1921, 1926 and 1930. Each case was operated on by a different surgeon. Four of the cases were females. The remaining case in a male is unusual. Crile states that he has never seen it occur in the male. The patients' ages were 18, 47, 36, 14 and 30.

*Type of Operation and Specimen Removed.*

| *Case.* | *Scope of Operation.* | *Nature of Specimen Removed.* |
|---|---|---|
| 1. | Both lobes of thyroid removed. Isthmus left. | Cystic adenoma. |
| 2. | Total thyroidectomy. | Multiple adenoma with calcareous degeneration. |
| 3. | One lobe had been removed eleven years earlier. Wedge resection of remaining lobe now done. | Parenchymatous goitre. |
| 4. | Wedge resection of two-thirds of each lobe. | Adenoma; no parathyroids found in specimen. |
| 5. | Hemithyroidectomy. | Multiple adenomata. |

The above table shows that the amount and method of removal differs in each case. It is difficult to condemn all these operations as not being properly performed. The operators were all full surgeons of the Hospital, and therefore not inexperienced in technique. On account of the more radical nature of the excision required, we should expect tetany to be more frequent after operations on exophthalmic goitre or malignant growth. Yet in none of these cases was hyperthyroidism a marked feature, and pathologically four were adenomatous and one parenchymatous. But tetany is not confined to the above types of operation.

C. H. Mayo[4] reports a case following enucleation of cystoadenomata. One of Von Eiselsberg's[3] patients had tetany after ligature of the four thyroid arteries.

Although tetany cannot always be avoided, every precaution must be taken to diminish the risk of its occurrence. First, complete removal of the parathyroids should be avoided, by doing only a partial or subtotal thyroidectomy. Berry[8] considers total thyroidectomy to be an unjustifiable operation. Even with this precaution we cannot be certain. Cases 3 and 4 were wedge resections and in Case 5 only one lobe was removed. Yet Du Bois has removed two normal parathyroid glands, from a patient with osteitis fibrosa, without producing tetany.

Can we explain the occurrence of tetany in these cases as resulting from irregular distribution of the parathyroids? Wellbrook,[5] examining 1,056 bodies, found supernumerary parathyroids in 7·7 per cent. These supernumerary glands may lie on the anterior surface of, or on the isthmus of, or embedded in the substance of, the thyroid. I can find no record of the percentage of cases in which the glands are absent from their normal position.

Secondly, the parathyroids lie in pairs near the distribution of the branches of the inferior thyroid arteries and are supplied by these vessels. Tetany may be due to crushing by artery forceps or by tying off this blood supply. Halstead, to avoid this, advises ligaturing the branches inside the capsule of the thyroid.

Thirdly, we have destruction of the parathyroids by scar tissue. This appears to be the most serious cause, as the parathyroids may be permanently destroyed, leading to chronic tetany. It usually follows from a second or third operation. There is a long interval after operation before tetany sets in. Thus in the case quoted by Aub, symptoms did not start until two months afterwards.

*Symptoms.*

The onset of tetany may occur from six hours up to four months after thyroidectomy. In these five cases it started at 12 hours, 24 hours, 24 hours, 7 days and 3 days respectively after operation.

There are two stages in the symptoms of tetany. The most acute phase, with spasm of the limbs, is obvious. The elbows and wrists are flexed and the hand pronated. The thumb is opposed to the palm, the knuckle joints are flexed, and the other finger joints extended, forming the so-called "accoucheur's hand." In the lower extremities the hips and knees are kept extended, the feet and toes being in plantar flexion. The distribution of the contractures varies in different cases. Thus in Case 1 the left foot, but not the right, was affected. In Case 4 the right hand was more affected than the left. In Case 5 the contractures in the feet did not start until some hours later than in the hands. Severe pain is complained of.

In the alternate phase there is no pain. The patient complains of a feeling of discomfort, of pins and needles, of stiffness and weakness, expecially in the forearm or calf. These are warning symptoms that the neuro-muscular system is in an irritable state.

That these symptoms are genuine may be supported by various signs. Chvostek showed that tapping the facial nerve set up spasm of that side of the face. Trousseau activated the latent spasm in the extremities by applying compression to the proximal end of the limb. Hoffman, by pressing over the nerve trunks, for example the ulnar at the inner side of the elbow, produced severe pain, thus demonstrating that the sensory nerves were also affected. Erb showed irritability of the motor nerves by applying the galvanic current. This gives a greater closing contracture at the anode than at the cathode.

Apart from a warning of the onset of the more acute stage, these signs are of importance as showing the necessity of continuing treatment and avoiding relapses.

*Treatment.*

(*a*) *Calcium.*—In 1909 MacCallum first showed experimentally that hypocalcæmia was associated with parathyroid tetany, and that injections of calcium into the blood relieved the muscular spasms. With regard to dosage by mouth, Hunter[6] advises up to 30 grammes daily. In normal individuals the rise in blood-serum calcium, after a dose of 10 grammes, is only 14 per cent. Hunter was able to control the blood-serum calcium of his patient, who had suffered from chronic tetany for twenty-two years, by giving 10 grammes of calcium daily. If the symptoms are severe, with laryngeal spasms or convulsions, he advises intravenous injection of 20 c.c. of a 5 per cent. solution of calcium chloride. Aub states that similar intravenous doses will relieve other types of spasm, such as renal or gallstone colic.

Four of the cases in my series were treated with calcium.

In Case 1 calcium lactate 60 grains was given by mouth four-hourly. The painful spasms ceased in three hours. Contractures of the hands lasted fifteen hours.

In Case 2 calcium lactate 20 grains was given by mouth thrice daily. This patient also had thyroid extract 1 grain twice a day. Contractures of hands lasted several days, but the patient was fit to go home on the fourteenth day after operation.

In Case 4 calcium bromide 10 grains was given by mouth thrice daily. The patient had completely recovered by the end of five days.

In Case 5 intramuscular injection of calcium chloride 1 grain relieved the pain for forty minutes, but the contractures were still present. The injection was repeated three hours later, parathyroid 1 grain also being given by mouth. Relief followed for another four hours, when painful spasms returned. An intravenous injection of calcium chloride 2 grains, with a subcutaneous injection of parathyroid 1/10th grain, was then given. Pain was relieved in forty-five minutes. Calcium lactate 30 grains, and parathyroid 1 grain, were given four-hourly by mouth, and continued for seven days. All symptoms had ceased by the sixth day from the onset.

The doses by mouth in the above cases varied from 30 to 360 grains daily. These cases show that the pains can be controlled whether the calcium be given by mouth, intramuscularly, or intravenously. Case 5 shows that, as we should expect, the intravenous route gives relief more rapidly.

(*b*) *Parathyroid.*—In 1896 Halstead attempted to treat tetany with sheep's parathyroid. In 1924 Collip[7] demonstrated that the active extract of parathyroid, when taken by the mouth, is rendered inert by the digestive juices. He therefore isolated an active extract, or hormone. This extract also is destroyed by trypsin and pepsin. It is therefore given subcutaneously. He showed experimentally that his parathormone, thus given, produces a rise in the blood serum calcium level. As an overdose may cause hypercalcæmia, the dosage must be controlled, by estimating the blood-serum calcium.

Hunter[6] has shown experimentally the results of parathormone therapy. He performed total thyro-parathyroidectomy on a dog. The animal had violent tetany in sixteen hours after operation. An intramuscular injection of fifteen units of parathormone was then given. Within three hours the dog became normal, and the tetanic symptoms had all disappeared. Hunter states that more severe cases of tetany require parathyroid extract in addition to calcium. Crile[1] reports the treatment of five cases of acute post-operative human tetany by parathormone. Relief of their symptoms was experienced within twenty minutes, after an intravenous injection of 1 c.c. of Collip's parathormone. Three doses of 2 c.c. to each case resulted in complete recovery.

In my series parathyroid was only given in one case, and was then combined with calcium.

Another method of giving parathyroid is by re-implanting the parathyroids, removed at the operation, either into the sterno-mastoid muscles or into the rectus abdominis. The results have not been wholly satisfactory. It is not always easy to find these small bodies measuring six by four millimetres. Lisser and Shephardson[9] grafted parathyroid from another patient into the rectus. Five months later, at post-mortem, no remains of the graft could be found.

If parathyroid extract merely acts by drawing calcium from the bones, what advantage has it over direct calcium therapy ? Chiefly in cases of chronic tetany, which fail to respond to calcium. Chronic tetany differs from the acute form, not so much in its onset, as in its progressive character. The symptoms are similar to acute tetany, but in addition pharyngeal, laryngeal, and bronchial spasms may supervene, accompanied by convulsions and epileptiform fits. The symptoms may persist for years ; in Aub's [6] case for nine years, and in Hunter's [6] case for twenty-two years. Opacities of the lens may develop, leading to cataract.

As none of my cases had chronic tetany, I venture to quote from other clinical sources.

Lisser and Shephardson's[9] case of chronic tetany followed the removal of a large adenomatous goitre. At operation only a minute portion of thyroid tissue was left over the isthmus. Presumably all the parathyroids were removed at the time of operation, as three were found then in the specimen, and at the post-mortem no further parathyroid tissue was found.

Although this patient was given calcium lactate 3 grammes daily by mouth, tetany still occurred, but was controlled for the time being by Collip's parathormone. Unfortunately, the dose required to be steadily increased. Starting with 12 units, by the end of eight months 100 units were required. The dose was finally raised to 160 units a day. Epileptiform attacks occurred, and the patient died of staphyloccal sepsis at the end of a year.

Aub's [6] case of chronic tetany was in a girl aged 17, with hyperthyroidism. Two operations were performed within a year, constituting total thyroidectomy. Tetany started two months after the second operation. Nine years later this patient still had tetany, and the blood-serum calcium was only 4·3 mgm. per 100 c.c. Fifteen units parathormone given daily raised the blood-serum calcium to 6·7 mgm., and attacks ceased. The effects of parathormone gradually diminished in spite of raising the dose to 100 units. Tetany associated with bronchial and laryngeal spasm and unconsciousness supervened, from which the patient died.

Thus in both these cases the primary success with parathormone was later counteracted by the patient's tolerance.

(*c*) *Thyroid.*—Aub[6] and his co-workers claim to have shown that the thyroid hormone stimulates calcium excretion more than parathyroid extract. In their case of post-operative tetany, treated with thyroid extract by mouth, and thyroxin intravenously, the blood calcium in two weeks rose from 6·7 to 11·9 mgms. per 100 c.c. This rise was maintained for six weeks after treatment had been stopped.

In Case 3 of my series thyroid extract 5 grains was given by mouth daily. The spasms ceased in five days, but were intermittent during this period. The patient was able to go home on the eighteenth day after operation. No other treatment was given.

Hunter's[6] experiments on animals do not fully support Aub's case. He performed total thyro-parathyroidectomy on cats and dogs. He then gave each animal the smallest dose of parathormone to keep it free from tetany, and injected thyroxin. As a result all the cats died of tetany. The dogs survived, but the blood-serum calcium dropped and tetany reappeared ten days after the thyroxin had been given. The advisability of using thyroid to treat tetany is, therefore, still an open question.

### CONCLUSIONS.

1. Tetany may follow thyroid operations, whether properly or improperly performed.

2. In the majority of cases the symptoms can be cured by calcium.

3. In the remaining cases the addition of parathyroid or thyroid is helpful, but further experimental work is still required.

In conclusion, I wish to thank my colleagues at the Bristol General Hospital for their cordial assistance in the preparation of this paper.

REFERENCES.

[1] Crile, *Endocrinology,* 1925, ix. 301.
[2] Dunhill, *Brit. Med. Jour.,* 1924, i. 5.
[3] Von Eiselsberg, *Handb. prakt. chir.*
[4] Mayo, C. H., *Keen's Surgery,* vi. 332.
[5] Wellbrook, *Jour. Amer. Med. Assoc.,* 1929, xcii. 1,821.
[6] Hunter, D., *Lancet,* 1930, i. 897.
[7] Collip, *Jour. Biol. Chem.,* 1925, p. 395.
[8] Berry, *Brit. Jour. Surg.,* 1921, viii. 413.
[9] Lisser and Shephardson, *Endocrinology,* 1929, xiii. 427.

